# Hepatitis C Infection and Treatment among Injecting Drug Users Attending General Practice: A Systematic Review and Meta-Analysis

**DOI:** 10.3390/ijerph20085569

**Published:** 2023-04-18

**Authors:** Meera Tandan, Shane Dunlea, Gerard Bury

**Affiliations:** General Practice, School of Medicine, University College Dublin, D04 N2E5 Dublin, Ireland

**Keywords:** hepatitis C, injecting drug users, general practice, treatment, primary care, systematic review

## Abstract

Background: The care provided in general practice to intravenous drug users (IDUs) with hepatitis C (HCV) extends beyond opioid substitution therapy. An aggregated analysis of HCV service utilization within general practice specifically related to diagnosis and treatment outcomes remains unknown from previous literature. Aims: This study aims to estimate the prevalence of HCV and analyze data related to the diagnosis and treatment-related outcomes of HCV patients with a history of intravenous drug use in the general practice setting. Design and setting: A systematic review and meta-analysis in general practice. Methods: This review included studies published in the following databases: EMBASE, PubMed, and Cochrane Central Register of Controlled Trials. Two reviewers independently extracted data in standard forms in Covidence. A meta-analysis was done using a DerSimonian and Laird random-effects model with inverse variance weighting. Results: A total of 20,956 patients from 440 general practices participated in the 18 selected studies. A meta-analysis of 15 studies showed a 46% (95% confidence interval (CI), 26–67%) prevalence rate of hepatitis C amongst IDUs. Genotype information was available in four studies and treatment-related outcomes in 11 studies. Overall, treatment uptake was 9%, with a cure rate of 64% (95% CI, 43–83%). However, relevant information, such as specific treatment regimens, treatment duration and doses, and patient comorbidities, was poorly documented in these studies. Conclusion: The prevalence of HCV in IDUs is 46% in general practice. Only ten studies reported HCV-related treatment outcomes; however, the overall uptake rate was below 10%, with a cure rate of 64%. Likewise, the genotypic variants of HCV diagnoses, medication types, and doses were poorly reported, suggesting a need for further research into this aspect of care within this patient group to ensure optimal treatment outcomes.

## 1. Introduction

Hepatitis C virus (HCV) causes both acute and chronic forms of hepatitis. More than 170 million people have been diagnosed with HCV worldwide, with 71 million people suffering from the chronic form of the disease [1]. The prevalence rate in the European Union region is 1.5%, with the highest rates existing in the Eastern Mediterranean (2.3%) [1]. There are large disparities in HCV infection rates between different sections of the population, although injecting drug users (IDUs) have been shown to represent a significant proportion internationally. Infections rates in this group has been shown to be between 13 to 84% in various national populations [2]. Those suffering from chronic HCV infection are at an increased risk of developing severe and potentially fatal liver diseases such as liver cirrhosis or hepatocellular carcinoma in the later stages of life [3]. Estimates for the proportion of mortality from hepatocellular carcinoma and chronic liver disease attributable to HCV are scarce across countries in the EU. However, overall estimates from 2015 show that 55% of liver cancer deaths, 44.7% of cirrhosis, and numerous other chronic liver disease deaths could have been attributed to hepatitis B and HCV infections in the EU/EEA region [4]. Liver disease-related mortality is 7.5% in IDUs with an HCV infection [4]. Human immunodeficiency virus (HIV) is an additional complication in this population, with historical infection rates as high as 60% among some IDU populations [5].

General practice (GP) is the first point of patient contact with the healthcare system in many countries and provides primary, personalized, and ongoing care to individuals and families in these communities. The care provided in general practice is sensitive to local community issues which often include roles as patient advocates [6]. The focus of the care provided to this patient group includes issues related to drug use and blood borne viral disease, but it extends far beyond this. General practice provides a holistic patient-centred system of care that encompasses all aspects of the physical and psychological wellbeing of this patient group, part of which entails developing and maintaining significant long-term relationships between doctors and patients.

Of the total number of patients with hepatitis C (HCV) who visited their GP doctor, the proportion of intravenous drug users (IDUs) was 76% in the United Kingdom (UK) and 70–80% in Ireland. [7,8,9]. However, HCV infection among IDUs is thought to remain underdiagnosed, which poses a significant health risk to both IDUs themselves as well as their injecting and sexual partners [10].

The treatment of HCV was revolutionized in 2011 with the introduction of oral direct-acting antivirals (DAAs) which have shorter treatment durations, fewer side-effects, and higher levels of patient acceptability than other medications [11,12,13]. Before DAA, pegylated interferon injection plus ribavirin oral administration was the conventional treatment and had a treatment success rate between 42–65% and poor tolerability due to adverse side effects [14,15].

The services provided by general practice for this patient group include the screening and diagnosis of HCV and other blood borne viral infections. They also include the evaluation, investigation, and treatment of healthcare issues both related and unrelated to IDU and HCV as well as patient referral to specialist care as appropriate; in some cases, DAA-based treatment regimens may be delivered in general practice.

It has previously been shown that IDUs with HCV on OST achieved a sustained virological response (SVR) rate of 71% compared to patients outside this cohort in a GP setting. This demonstrates the key role of OST in successful treatment uptake and outcomes in this group [16]. Other studies have also shown that IDUs with HCV have a lower probability of receiving antiviral therapy than non-IDU HCV patient groups [17,18]. One of the barriers to successful HCV treatment in IDUs is their often chaotic lifestyles, which often involve homelessness, alcohol and illicit drug abuse, social isolation, poor medication compliance, and various forms of social stigma. The fear and stigma of investigations such as HIV testing have been shown to be an additional consideration in this patient group [7,8,9]. Those diagnosed with HCV and initiated on or referred for treatment often display high rates of dropout due these factors which also include co-existing psychiatric illnesses and other psychosocial problems [17,19].

This study aims to investigate and estimate (i) the prevalence of hepatitis C amongst IDUs, (ii) diagnostic actions, (iii) antiviral treatments, and (iv) cure rates, in a general practice setting, with inclusion of a meta-analysis where appropriate.

## 2. Materials and Methods

### 2.1. Data Source and Search Strategy

This is a systematic review and meta-analysis conducted following the review guidelines provided in the *Cochrane Handbook for Systematic Reviews of Interventions* [20]. We limited our search to EMBASE, PubMed, and Cochrane Central Register of Controlled Trials. The initial database search was performed between the 1st of October and the 18th of November 2020, with an updated search carried out on 1 March 2023. The search terms used were: “hepatitis C”, “hepacivirus”, “general practice”, “drug users”, “intravenous drug users”, and “antiviral agents”. Controlled vocabulary terms (MesH term and Emtree entries) along with Boolean operators (OR, AND, and NOT) were combined to make a search strategy.

### 2.2. Screening and Eligibility

**Figure 1:**
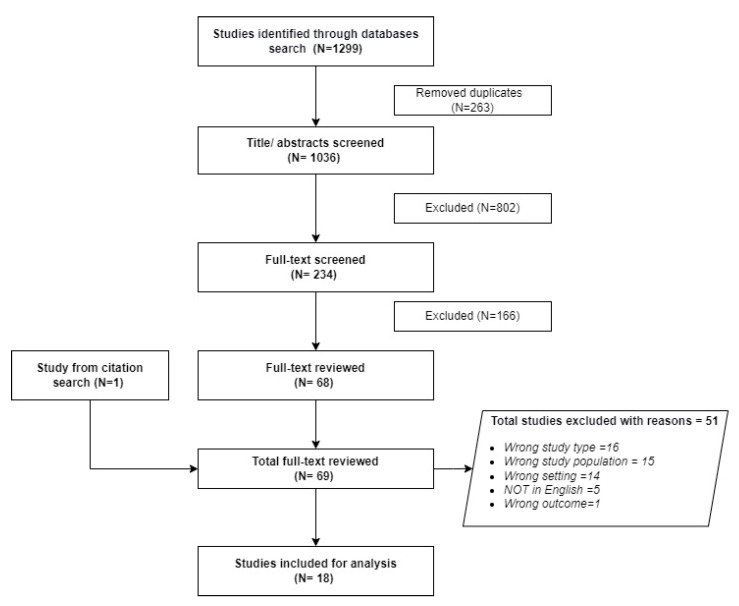
PRISMA—selection of studies.

We followed the Preferred Reporting Items for Systematic Reviews and Meta-analyses (PRISMA) guidelines [21] to select studies (Figure 1). Two authors (M.T. and S.D.) independently searched for relevant literature using the search terms and criteria. All the identified studies were uploaded to a bibliographic management software (EndNote X9 for Windows). All duplicate studies were removed, and the titles and abstracts of included studies were screened for eligibility. All eligible studies were transferred to Covidence, an online review management software. In Covidence, a full-text analysis of all included studies was performed. The agreement score (Cohen Kappa) between the two reviewers was 81% in the first phase of full-text eligibility screening. Consensus was reached on the outstanding remaining studies with input from a third reviewer (G.B.).

### 2.3. Inclusion and Exclusion Criteria

A study was considered eligible if it was conducted in general practice, had hepatitis C positive IDUs as its study participants, and reported on HCV prevalence and treatment outcomes.

The studies excluded were systematic reviews or opinion articles, editorials, pharmacological studies, and studies conducted in a primary care setting other than GP practices, such as in methadone clinics, opioid treatment centres, and care centres providing services through the integration of specialist centres with primary care delivery at the community level. Studies not published in English were also excluded from the analysis.

### 2.4. Quality Assessment

We used the standard checklist produced by the US National Health Lung and Blood Institute for assessing observational cohort and cross-sectional studies to judge the quality of cohort and cross-sectional studies [22], while Cochrane’s tools for assessing risk of bias [23] were used to assess the quality of randomized controlled trials. The outcome of assessed bias was recorded as high risk, low risk, or unclear. The focus of the quality assessment was: (i) objectiveness and clarity of the research questions, (ii) study population definition and clarity, (iii) inclusion and exclusion criteria, (iv) sample size and power justification, (v) use of valid and reliable measures across study participants, and (vi) consideration of the potential confounding variables for cohort and cross-sectional studies. For the controlled trials, the assessment elements were: (i) appropriate randomization, (ii) group consistency in the intervention and control arms, and (iii) baseline and blinded assignments of the groups, as well as all other elements used to assess cross-sectional studies.

### 2.5. Study Outcomes

Outcome data extracted from the eligible studies included: treatment effectiveness in terms of cure rates, SVR rates, treatment adherence rates, reinfection rates, HCV-related comorbidities, details of medication regimes (type, duration, and dosage), and any adverse drug events. Information such as SVR, adherence, reinfections, and adverse drug events were never reported in the studies.

### 2.6. Data Extraction and Analysis

Data extraction was independently performed in Covidence by M.T. and S.D. Both data extraction and quality assessment were customized to collect information on our variables of interest. The final data entry was conducted after pretesting three studies. The variables collected included: year of publication, study population, study sites, number of practices, demographic characteristics, IDUs and the number of HCV infections, information on OST and treatment of HCV infections, drugs and duration of treatment, the number of patients cured, and chronic conditions.

**Figure 2:**
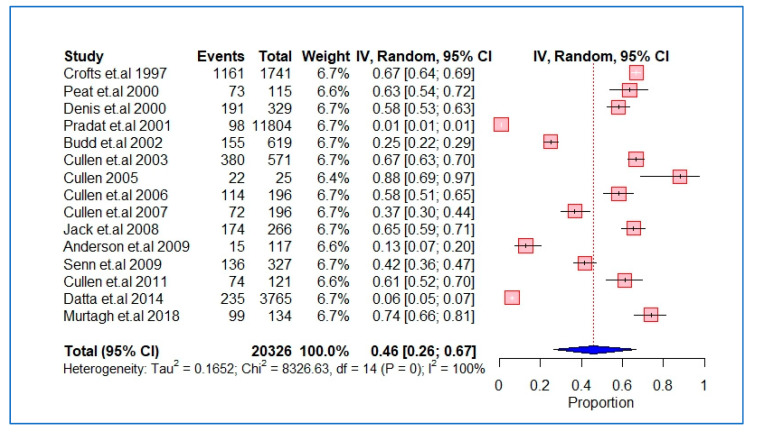
Pooled prevalence of HCV infection among IDUs in GP practice.

**Figure 3:**
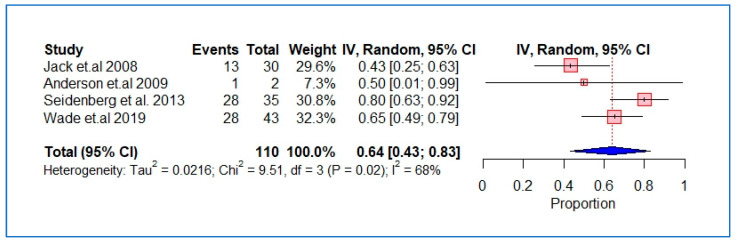
Pooled proportion of cure rate in HCV patients with a history of intravenous drug use.

**Figure 4:**
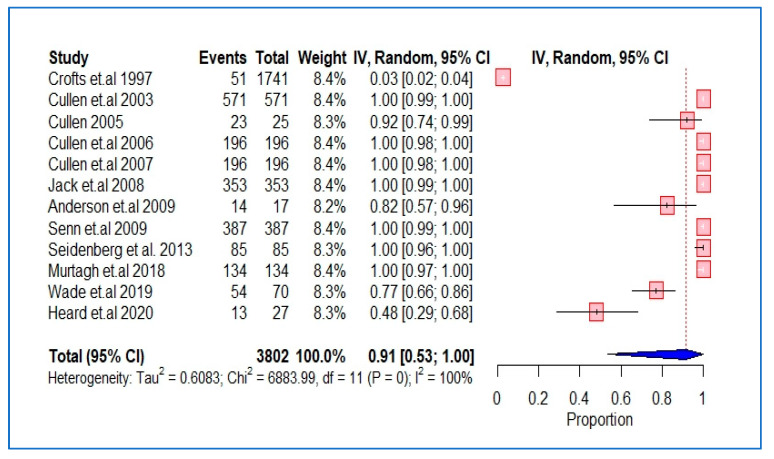
Pooled proportion of OST users in GP practices.

The characteristics and findings of the studies included were summarized and structured using tables and figures where applicable. We performed a meta-analysis of the studies using the DerSimonian and Laird random-effects model with inverse variance weighting [24]. A forest plot was used to show the pooled estimates, where the diamond shape represents the overall effect estimate and the small boxes with horizontal lines show the effect estimates for individual studies (Figure 2, Figure 3 and Figure 4). The length of the horizontal lines and width of the diamond illustrate the confidence interval. Statistical heterogeneity was assessed using the chi-square test of heterogeneity and the I^2^ statistic for measuring inconsistency, with higher I^2^ values indicating higher heterogeneity. Based on Cochrane’s recommendations, we considered I^2^ values of 30–60% as indicating moderate heterogeneity in the studies and values above this range as indicating substantial heterogeneity. The meta-analysis and risk of bias analyses was performed using the “Meta” package in R version 4.0.3 (accessed on 10 October 2020).

## 3. Results

### 3.1. Selection of Included Studies

Figure 1 shows the 1063 studies selected after removing the 263 duplicates from the 1299 studies extracted from the database. We retained 69 studies for full-text review after excluding 166 studies following title and abstract screening. Finally, data were extracted from a total of 18 studies [7,8,9,16,25,26,27,28,29,30,31,32,33,34,35,36,37,38] after 51 studies were excluded following a full-text review (Figure 1: PRISMA). The reasons for exclusion were: (i) wrong study type (16 studies), (ii) wrong study population (15 studies), (iii) wrong setting (14 studies), (iv) study not in English language (5 studies), and (v) wrong study outcome (1 study).

### 3.2. Characteristics of the 18 Included Studies

A total of 20,956 participants were enrolled across 440 GP practices between 1997 to 2020 with the study duration ranging between 1.5 and 89 months; 13/18 (72%) studies were published before 2012 and only two explored the role of DAA treatment in general practice (Table 1). The majority of the studies were from the UK (6) and Ireland (5) and the rest were from Australia (3) and other European countries. Almost all studies published in Ireland were from the same research group. Most studies reported on GP sites providing OST care. Of the 18 studies selected, five were controlled intervention studies. Males represented 64% of the study participants, and the average age was between 25 and 47 years.

### 3.3. Prevalence of HCV among Patients with a History of Intravenous Drug Use

Of the 18 studies, 15 [7,8,9,25,27,28,29,30,32,33,34,35,36,37,38] were included in the meta-analysis of the prevalence of HCV among IDUs (Figure 2). Measurement of seroprevalence and/or screening of HCV infection was listed as one of the main objectives in 10/15 studies [7,8,27,28,29,32,33,34,35,37,38]. Overall, the prevalence of HCV infection among IDUs in GP was 46% (95% confidence interval (CI), 26–67%); however, the studies had significant heterogeneity (I^2^ = 100%, *p* = 0.00). A subgroup analysis comparing studies published before and after 2010 did not show any significant change in the prevalence of HCV among IDUs (Figure S1).

### 3.4. HCV Diagnosis, Treatment, and Cure Rates

Information relating to specific genotypes was reported in four studies [16,25,31,36] and treatment-related outcomes was reported in 11 studies [9,16,26,27,28,29,30,31,33,36,37]. Genotype 1 and genotype 3 were noted in all four of these studies (Table 2). Only one study, which involved 70 participants, reported the duration of HCV infection, with a mean duration of 19 years [30].

Eleven studies reported information related to treatment outcomes (Table 2). A total of 9% (174/1954) of patients were treated for their HCV infections. This treatment rate was above 60% in two studies [26,31], 40% in one study [16], and below 5% in six studies [27,28,29,30,33,37].

Only four studies [16,27,31,37] reported specific medication regimes while drug information, including the doses and duration relating to genotypic information, was reported in only two studies [16,31]. Interferon was prescribed in studies conducted in 2000 [37] and 2005 [27]. Peginterferon plus ribavirin was prescribed for 48 weeks in a study from 2013 [16], while the most recent study published in 2019 [31] used DAAs in combination with ribavirin for 12 weeks (Table 2).

The number of participants treated and cured was available for four studies [16,31,33,36]; hence, a meta-analysis was run to estimate the pooled ‘proportion of cure’ rate. The estimated cure rate was 64% (95% CI, 43–83%; Figure 3). The included studies had substantial heterogeneity (I^2^ = 68%, *p* = 0.02). The cure rate was higher in the studies published after 2010 (72%) compared to those published before (43%) (Figure S2).

### 3.5. Opiate Substitution Therapy (OST)

Of the 14 studies which reported the OST status of study participants, 13 involved general practice centres which provided this service (Table 1). The OST status of the participating practices was unclear in four studies; these four studies contributed 279/440 (63.4%) of all practices and 16,303/20,956 (77.8%) of all patients involved in this review [7,32,35,38]. The meta-analysis of these 12 studies estimated an overall proportion of 91% (95% CI, 53–100%) of patients on OST (Figure 4). The differences between the studies reported before and after 2010 were not significant (Figure S3).

### 3.6. Chronic Conditions

**Figure 5:**
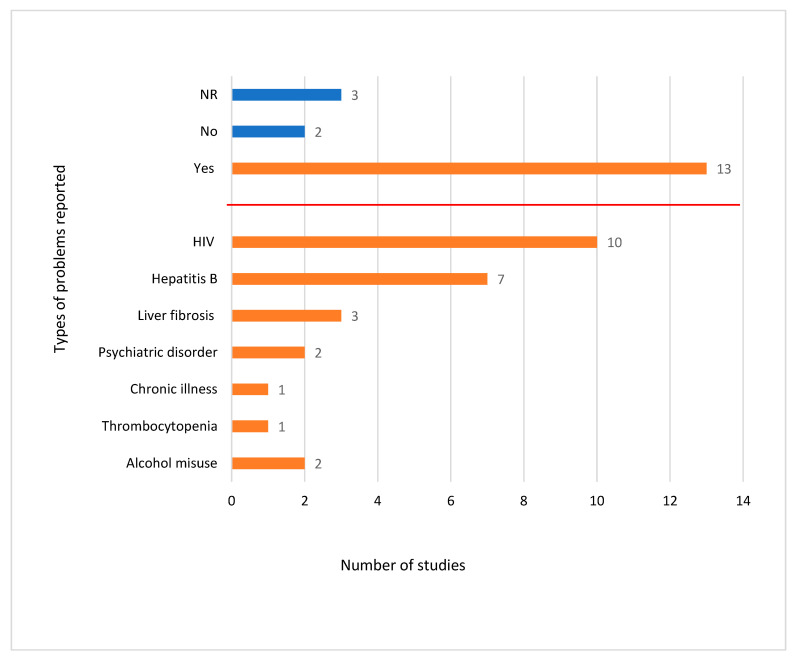
Number of studies reporting chronic health issues and alcohol misuse (*n* = 19).

Thirteen studies [7,8,9,16,25,27,28,30,31,32,35,36,37] published information related to concomitant medical conditions and alcohol misuse amongst study participants (Figure 5). HIV co-infection rates were reported in 10 studies [7,8,9,16,25,30,32,35,37], with an overall prevalence of 10% (427/6201). The rates of psychiatric disorders and alcohol misuse were reported in two studies [16,25], and the overall prevalence for these conditions was above 70% (332/472) and 20% (104/472), respectively.

### 3.7. Risk of Bias Assessment

An assessment of the risk of bias in the selected studies showed that 50% of the studies were ranked as having a high risk of bias related to the criteria of sample size and power calculation. The risk of bias was categorised as unclear in 80% of the studies when assessed for their measurement and adjustment for key confounding variables (Figure S4). Of the five included randomized trials [28,29,31,33,36], 30% of the studies had a high risk of bias related to the blinding of study participants. The risk of bias was classified as unclear in 75% of these studies in relation to the groups having similar baseline characteristics, 70% in relation to the blinding of study participants, and 60% each in relation to describing the study as an RCT for all outcomes assessed and randomization (not shown in the figure).

## 4. Discussion

### 4.1. Summary

It is notable that this systematic review identified only 18 studies fitting the inclusion criteria of being based in general practice and involving hepatitis C positive IDUs. Twelve of the 18 studies were dated from before 2011 (when the introduction of DAAs made a major impact on treatment options). A limited number of these studies reported on the treatment outcomes of participants, and the quality of the studies was highly variable.

General practice has the potential to contribute significantly to the holistic, long-term care of the wide range of health and psychosocial issues affecting this group of patients and may also play a key role in contributing to the targeted treatment of hepatitis C. Although general practice in many healthcare systems is likely to be performing many such roles already, there is a real dearth of research data exploring this aspect of care.

This review identified 18 studies that reported on the care of hepatitis C infections in IDUs which were based in a GP-based primary care setting. All but four of the included studies [34,35,37,38] were from the 1990s. Adequate information that allowed for the estimation of the prevalence of hepatitis C amongst IDUs utilizing care was available in 15 studies, with the pooled prevalence rate found to be 46%. Apart from one randomized trial in 2019 [31], none of the studies reported the duration of HCV infection amongst study participants. Diagnostic information, primarily the specific HCV genotype, was reported in four studies, and treatment-related outcomes were reported in 11 studies.

The majority of the studies reported treatment uptake rates below 5%, except three studies which had rates between 41 and 74% [16,26,31]. The higher percentages could be due to fact that the primary objectives of these three studies were treatment uptake and cure rates. In contrast, the primary objectives of the other studies were prevalence rates, risk factor identification, and treatment care processes. Other reasons for low treatment uptake may include the difficulty of follow-up in this population of participants and a low level of awareness regarding treatment options amongst clinicians. Another confounding factor is likely the need for issues such as alcohol misuse and HIV treatment to be appropriately managed before commencing hepatitis C treatment in many cases [39,40]. However, further independent research is required to understand the perspectives of both care providers and IDUs related to treatment uptake rates. Similarly, information related to the specific hepatitis treatment regimens used was available for only four studies. This precluded us from performing a meta-analysis to compare the effectiveness of various treatment regimes. Hence, it could be an area for exploration in future primary care research initiatives.

An analysis looking at the available genotypic information showed that more genotype 1 patients received treatment in the Seidenberg et al. study [16] and more genotype 3 patients received treatment in the Jack et al. study, which likely reflects local seroprevalence rates [36]. The specific drug regimens prescribed to treat study participants were reported in four studies, and dose and duration data were reported in two of these. Between 2002 [37] and 2005 [27], two studies reported the use of interferon treatment by injection, as interferon was the drug of choice during that period. As previously discussed, the treatment options for hepatitis C have developed and evolved over the years. Previous studies have shown that DAA drugs have a higher sustained virological response and fewer side effects compared to their predecessors. The shorter duration of DAA treatment and oral route of administration means that they are less burdensome to both patients and physicians. The number of patients cured was reported in four studies, with an estimated cure rate of 64%. However, the proportion obtained in the meta-analysis was not sufficient to draw a definitive conclusion because of the small number of studies and the sample size of 110. Specifically, Anderson et al. [33] had only two patients treated in their study.

Among the studies that reported on OST use, the majority of the GP practices included in these studies were found to provide OST services, and 91% of the IDUs diagnosed with hepatitis C were found to be on OST. The rate of reporting related to participants’ medical co-morbidities, such as specific diagnoses and quantitative data, was very poor. In studies where such data were reported, it was often unclear. Specific conditions listed included HIV co-infection (10 studies), hepatitis B co-infection (7 studies), liver fibrosis (3 studies), and psychiatric disorders (2 studies). Significantly more patients in this cohort were noted to have been diagnosed with a psychiatric disorder than with an HIV co-infection. In one study, psychiatric disorders, particularly depression, were found to be associated with significantly increased levels of active drug misuse [41].

### 4.2. Strengths and Limitations

In the meta-analysis, significant heterogeneity was observed among the included studies. To some extent, heterogeneity can be explained and overcome by subgroup analysis and sensitivity analyses; however, the lack of studies reporting treatment information did not allow us to perform subgroup and sensitivity analyses. The majority of the studies included were cross-sectional and retrospective cohort reviews designed to study seroprevalence and risk factors without any comparison group; hence, a comparison of the prevalence between different groups, such as age and gender, was not feasible. Even though treatment information was reported in 11 studies, information specific to cure rates, drugs, dose, and duration was missing from many studies, which precluded us from conducting a robust data analysis comparing different treatment types based on genotype information. Despite this, we tried to explore differences in the prevalence of HCV in IDUs, OST use, and cure rates by comparing studies published before and after 2010. The analysis showed no significant difference in the prevalence and proportion of OST use before and after 2010 among HCV-infected IDUs. The HCV cure rate was found to be higher in the studies conducted after 2011; however, there were only two studies included in each subgroup in the meta-analysis. In addition, 50% of the papers included were assessed as having a high of bias. Therefore, considering both the high heterogeneity and high risk of bias in these studies, the findings of this review should be interpreted cautiously.

The studies included in this review spanned the period from 1991 to 2018. Many of the studies (67%) were conducted during the period before DAA became widely available (2011). Even though the included studies were insufficient in number and power such that their findings cannot be generalized, our analysis showed encouraging progress in the level of care provided to IDU hepatitis C patients attending general practice after 2011. However, between the 1990s and 2010s, there were significantly fewer developments in the care of this cohort of patients. The causes of such limited development are difficult to identify based on the current evidence. The literature [42,43] suggests that during this period, novel antiviral therapies were still in their infancy and fewer therapeutic options suitable for use in the community were available. The introduction of pegylated interferon and ribavirin (with a 50% virological cure rate in generally adherent patients in 2001/2005) were indicators of a brighter future for hepatitis C care [42,43]. Our review indicates an increased level of activity since the subsequent introduction of DAA therapies and clearly establishes the need for high quality research to maximise the potential of such therapies in the community.

### 4.3. Comparison with the Literature

The prevalence and effectiveness of HCV treatment has been documented independently in the literature [41,42,43,44]. A systematic review and meta-analysis of the prevalence and treatment of hepatitis C among IDUs, particularly in general practice, has not been published yet. Our study showed a pooled prevalence of between 26 and 67% of IDUs diagnosed with hepatitis C infection in primary care. This finding is similar to the prevalence rates reported among IDUs in Iran [44] and Pakistan [45]; however, it is lower than the prevalence rate of 80% reported in the EU region [41]. The study in the EU region was not solely a study among IDUs, and the proportion was part of a subgroup analysis obtained from the general population. On the other hand, the study populations from Iran and Pakistan mostly involved patients from community drug treatment centres, with a few studies involving patients from secondary care.

Our study reported variation in hepatitis C treatment uptake, with an overall treatment uptake of 9% amongst this cohort of patients, which is similar to the treatment uptake reported in a study conducted in opioid-dependent patients from OST centres [46]. The proportion in our study was three times lower than the treatment uptake reported in a systematic review from the EU region; however, the setting in that study was not restricted to general practice care [47]. Even so, these studies report a significantly higher treatment uptake among IDUs utilizing specialist services based in primary care, with a significantly higher SVR also being reported [42]. The reasons for the differences between GP-only and specialist-based services may be related to different patient or service characteristics, differences in how the studies report their information, or other unspecified factors.

Our study estimated the reported HCV cure rate in GP to be between 43 and 80%, which differed from the cure rate of 19–88% reported by Lazarus et al. [47]. Once again, this difference could be due to the very low number of studies included in our review compared to other studies or to the difference in study location and settings.

## 5. Conclusions

A dearth of good quality research data exists in relation to the current and potential roles of general practice in the holistic care of IDUs with HCV. In particular, very limited data that explores the potential of DAAs in general practice exists, although data from primary care-based specialist services are promising. Further research aimed at exploring these issues is required in general practice.

Overall, the prevalence of HCV among IDUs in GP care was above 60%. In addition, OST appears to be an important element in care. As diagnostic and treatment information was reported in only a small number of studies, this made drawing concrete conclusions from the current study difficult. This study indicates the need for future research involving this target population to better inform the service requirements and resource allocation needed in general practices. Future research to identify the causes of low treatment uptake and cure rates will be essential and will help optimise treatment acceptance and compliance amongst this vulnerable patient group.

## Figures and Tables

**Table 1:** Characteristics of the included studies.

Study ID	Study Design	# of GP Sites	Are All Practices OST Centres?	Sample (Participants)	Study Period (Duration)	Sex (M/F)	Age (years)	Country	Main Outcome of the Study
Crofts et al. 1997 [34]	Retrospective cohort review	1	Yes	1741	January 1991–December 1995 (73 months)	1010/730	Mean 32.5	Australia	HCV seroprevalence and effect of methadone maintenance therapy on HCV control
Peat et al. 2000 [35]	Retrospective cohort review	1	NR	115	October 1999–February 2000 (5 months)	NR	NR	UK (Scotland)	HCV prevalence and referral to specialist treatment for infection
Denis et al. 2000 [37]	Cross-sectional, comparative	10	Yes	329	1995–June 1998 (NR)	224/85	Mean 25.9 Range 16–45	Belgium	HCV seroprevalence—associated risk factors and feasibility of treatment
Pradat et al. 2001 [38]	Cross-sectional	271 *	NR	11,804	May–October 1997 (6 months)	NR	NR	France	HCV seroprevalence
Budd et al. 2002 [32]	Cross-sectional	1	NR	619	January–May 2000 (5 months)	NR	NR	UK (Scotland)	HCV prevalence and associated risk factors
Cullen et al. 2003 [13]	Retrospective record review	42	Yes	571	NR (NR)	409/162	Mean 28	Ireland	HCV seroprevalence and associated factors
Cullen 2005 [27]	Cross-sectional	1	Yes	25	2002 (1 months and 2 weeks)	14/11	Mean 32	Ireland	Awareness and experience of HCV infection, investigation, and treatment
Cullen et al. 2006 [28]	Cluster randomized controlled trial	26	Yes	196	NR (6 months)	142/54	Mean 32.5	Ireland	HCV screening and evaluation of clinical guideline implementation and treatment outcome
Cullen et al. 2007 [30]	Cross-sectional	25	Yes	196	Early 2002 (NR)	142/54	Mean 32.2 Range 19–65	Ireland	HCV infection care process—testing, hepatology referral, HCV treatment, alcohol consumption
Jack et al. 2008 [36]	Clinical trial	2	Yes	353	February 2005–January 2008 (36 months)	256/26	Mean 34.7 Range 21–53	UK (England)	HCV diagnosis, feasibility of antiviral treatment and outcome
Anderson et al. 2009 [33]	Controlled intervention	2	Yes	117	November 2003–April 2004 (6 months)	180/241	NR	UK (Scotland)	HCV screening evaluation—test offer, uptake, and diagnosis
Senn et al. 2009 [25]	Retrospective cohort review	1	Yes	387	January 2002–May 2008 (89 months)	268/119	Median 38.5	Switzerland	Assessment of chronic HCV infection—viral load, genotypes
Cullen et al. 2011 [29]	Controlled intervention	16	Yes	422	February–October 2007 (9 months)	NR	Range 30–54	UK (Scotland)	HCV seroprevalence—test uptake, referral, management of PCR in intervention and control sites
Seidenberg et al. 2013 [16]	Retrospective cohort	1	Yes	85	January 2002–May 2008 (89 months)	52/33	Median 38.8	Switzerland	HCV treatment rate and sustained virological response rates between patients with and without drug dependency
Datta et al. 2014 [12]	Cross-sectional	6	NR	3765	August 2012–January 2013 (6 months)	NR	NR	UK (England)	HCV seroprevalence
Murtagh et al. 2018 [14]	Retrospective cohort-feasibility study	14	Yes	134	NR (NR)	96/38	Mean 43 Range 27–71	Ireland	HCV management—process and outcomes
Wade et al. 2019 [31]	Randomized controlled trial	13	Yes	70	November 2015–June 2018 (32 months)	52/18	Mean 47	Australia and New Zealand	HCV treatment: direct-acting antiviral treatment uptake and sustained virological response
Heard et al. 2020 [26]	Cross-sectional qualitative	7	No, only 5 out of 7	27	NR (NR)	18/9	Range 33–65	Australia	HCV management—barriers and enablers of direct-acting antiviral treatment
Total		440		20,956		2863/1580			

* GPs (assuming different practices), OST = opioid substitution therapy, NR = not reported, M= male, F = female, # = Number.

**Table 2:** HCV diagnostic and treatment-related information in the included studies.

Study ID	Study Reported HCV Treatment	Number Treated	Genotypes Detected (Treated)	Drugs by Genotypes and Duration
Denis et al. 2000 [37]	Yes	10	NR	Interferon
Cullen 2005 [27]	Yes	1	NR	Interferon
Cullen et al. 2006 [28]	Yes	6	NR	NR
Cullen et al. 2007 [30]	Yes	3	NR	NR
Jack et al. 2008 [36]	Yes	30	Genotype 1 (7), Genotype 3 (14), Genotype unknown (9)	NR
Anderson et al. 2009 [33]	Yes	2	NR	NR
Senn et al. 2009 [25] *	NR		Genotype 1 (43), Genotype 3 (34), Genotype 4 (9) *	
Cullen et al. 2011 [29]	Yes	4	NR	NR
Seidenberg et al. 2013 [16]	Yes	35	Genotype 1 (19), Genotype 3 (13), Genotype 4 (30)	Genotypes 1 and 4—once-weekly injections of peginterferon alfa-2a (180 μg) plus ribavirin (1000 mg or 1200 mg/day) for 48 weeks. Genotype 3—ribavirin 800 mg/day and peginterferon alfa-2a 180 μg/week subcutaneously for 24 weeks.
Murtagh et al. 2018 [14]	Yes	20	NR	NR
Wade et al. 2019 [31]	Yes	43	Genotype 1, Genotype 1a, and Genotype 3	Genotype 1—co-formulated paritaprevir 75 mg, ritonavir 50 mg, and ombitasvir 12.5 mg in two tablets daily plus dasabuvir 250 mg one tablet twice daily for 12 weeks. Genotype 1a—weight-based ribavirin; patients ≤75 kg received 1000 mg and patients ≥75 kg received 1200 mg daily for 12 weeks. Genotype 3—sofosbuvir 400 mg and daclatasvir 60 mg one tablet daily for 12 weeks.
Heard et al. 2020 [26]	Yes	20	NR	NR

* The numbers in the parentheses in column four represent only the genotypes detected, NR = Not reported.

## Supplementary Materials

The following are available online at https://www.mdpi.com/article/10.3390/ijerph20085569/s1, Figure S1: Meta-analysis of HCV prevalence by decades; Figure S2: Meta-analysis of HCV prevalence by decades; Figure S3: Meta-analysis of HCV prevalence by decades; Figure S4: Risk of bias summary of included studies.
