# Supplementary material for: Hepatitis C Infection and Treatment among Injecting Drug Users Attending General Practice: A Systematic Review and Meta-Analysis
Source: Int J Environ Res Public Health. 2023 Apr 18;20(8):5569. doi: 10.3390/ijerph20085569 (PMC10138322; doi:10.3390/ijerph20085569)

Figure S1- Meta-analysis of HCV prevalence by decades

Study or Subgroup	Events	Total	Weight	IV, Random, 95% CI
**Year = Until 2010**				
Crofts et.al 1997	1161	1741	6.7%	0.67 [0.64; 0.69]
Peat et.al 2000	73	115	6.6%	0.63 [0.54; 0.72]
Denis et.al 2000	191	329	6.7%	0.58 [0.53; 0.63]
Pradat et.al 2001	98	11804	6.7%	0.01 [0.01; 0.01]
Budd et.al 2002	155	619	6.7%	0.25 [0.22; 0.29]
Cullen et.al 2003	380	571	6.7%	0.67 [0.63; 0.70]
Cullen 2005	22	25	6.4%	0.88 [0.69; 0.97]
Cullen et.al 2006	114	196	6.7%	0.58 [0.51; 0.65]
Cullen et.al 2007	72	196	6.7%	0.37 [0.30; 0.44]
Jack et.al 2008	174	266	6.7%	0.65 [0.59; 0.71]
Anderson et.al 2009	15	117	6.6%	0.13 [0.07; 0.20]
Senn et.al 2009	136	327	6.7%	0.42 [0.36; 0.47]
**Total (95% CI)**		**16306**	**80.0%**	**0.47 [0.20; 0.75]**
Heterogeneity: $Tau^2 = 0.2601$; $Chi^2 = 7831.28$, df = 11 (P = 0); $I^2 = 100\%$				
**Year = 2011−2022**				
Cullen et.al 2011	74	121	6.6%	0.61 [0.52; 0.70]
Datta et.al 2014	235	3765	6.7%	0.06 [0.05; 0.07]
Murtagh et.al 2018	99	134	6.7%	0.74 [0.66; 0.81]
**Total (95% CI)**		**4020**	**20.0%**	**0.44 [0.02; 0.93]**
Heterogeneity: $Tau^2 = 0.2530$; $Chi^2 = 495.35$, df = 2 (P < 0.01); $I^2 = 100\%$				
**Total (95% CI)**		**20326**	**100.0%**	**0.46 [0.26; 0.67]**

Heterogeneity: $Tau^2 = 0.1652$; $Chi^2 = 8326.63$, df = 14 (P = 0); $I^2 = 100\%$

Test for subgroup differences: $Chi^2 = 0.01$, df = 1 (P = 0.94)

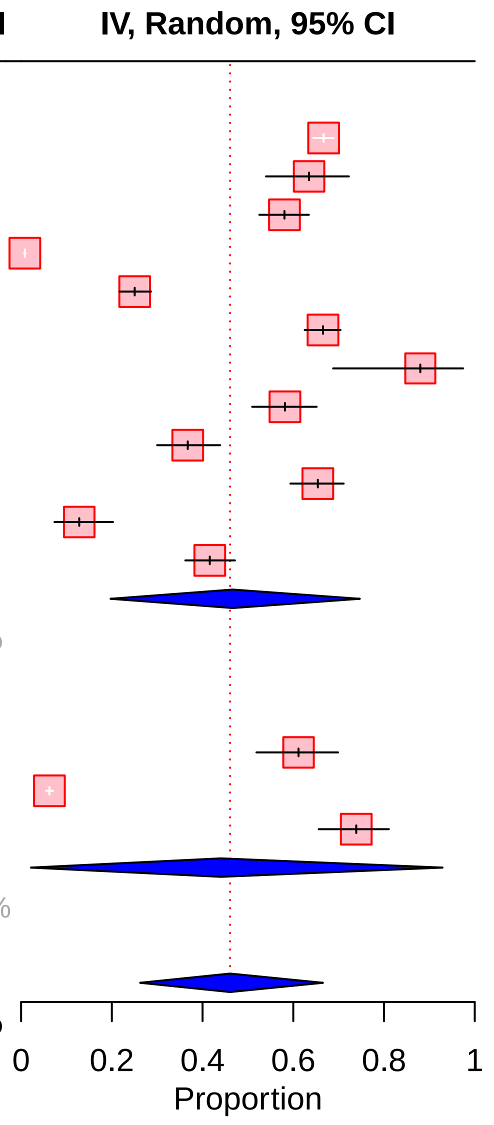

Figure S2 - Meta-analysis of HCV cure rate by subgroup decades

Study or Subgroup	Events	Total	Weight	IV, Random, 95% CI
**Year = Until 2010**				
Jack et.al 2008	13	30	29.6%	0.43 [0.25; 0.63]
Anderson et.al 2009	1	2	7.3%	0.50 [0.01; 0.99]
**Total (95% CI)**		**32**	**36.9%**	**0.43 [0.23; 0.64]**
Heterogeneity: $Tau^2 = 0$; $Chi^2 = 0.04$, df = 1 (P = 0.8440); $I^2 = 0\%$				
**Year = 2011 −2022**				
Seidenberg et al. 2013	28	35	30.8%	0.80 [0.63; 0.92]
Wade et.al 2019	28	43	32.3%	0.65 [0.49; 0.79]
**Total (95% CI)**		**78**	**63.1%**	**0.72 [0.57; 0.86]**
Heterogeneity: $Tau^2 = 0.0067$; $Chi^2 = 2.04$, df = 1 (P = 0.1530); $I^2 = 51\%$				
**Total (95% CI)**		**110**	**100.0%**	**0.64 [0.43; 0.83]**
Heterogeneity: $Tau^2 = 0.0216$; $Chi^2 = 9.51$, df = 3 (P = 0.0232); $I^2 = 68\%$				
Test for subgroup differences: $Chi^2 = 5.83$, df = 1 (P = 0.0157)				

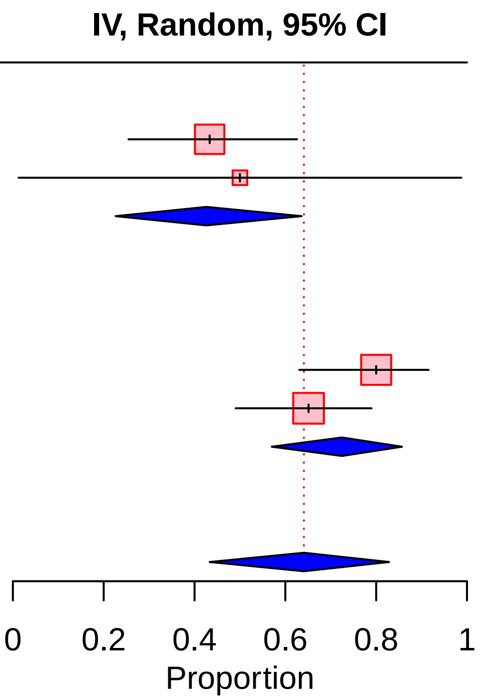

Figure S3: Meta-analysis of OST user by subgroup decades

Study or Subgroup	Events	Total	Weight	IV, Random, 95% CI
**Year = Until 2010**				
Crofts et.al 1997	51	1741	8.4%	0.03 [0.02; 0.04]
Cullen et.al 2003	571	571	8.4%	1.00 [0.99; 1.00]
Cullen 2005	23	25	8.3%	0.92 [0.74; 0.99]
Cullen et.al 2006	196	196	8.4%	1.00 [0.98; 1.00]
Cullen et.al 2007	196	196	8.4%	1.00 [0.98; 1.00]
Jack et.al 2008	353	353	8.4%	1.00 [0.99; 1.00]
Anderson et.al 2009	14	17	8.2%	0.82 [0.57; 0.96]
Senn et.al 2009	387	387	8.4%	1.00 [0.99; 1.00]
**Total (95% CI)**		**3486**	**66.7%**	**0.92 [0.43; 1.00]**
Heterogeneity: $Tau^2 = 0.6695$; $Chi^2 = 6504.69$, df = 7 (P = 0); $I^2 = 100\%$				
**Year = 2011−2022**				
Seidenberg et al. 2013	85	85	8.3%	1.00 [0.96; 1.00]
Murtagh et.al 2018	134	134	8.4%	1.00 [0.97; 1.00]
Wade et.al 2019	54	70	8.3%	0.77 [0.66; 0.86]
Heard et.al 2020	13	27	8.3%	0.48 [0.29; 0.68]
**Total (95% CI)**		**316**	**33.3%**	**0.89 [0.64; 1.00]**
Heterogeneity: $Tau^2 = 0.0945$; $Chi^2 = 86.21$, df = 3 (P < 0.01); $I^2 = 97\%$				
**Total (95% CI)**		**3802**	**100.0%**	**0.91 [0.53; 1.00]**

Heterogeneity: $Tau^2 = 0.6083$; $Chi^2 = 6883.99$, df = 11 (P = 0); $I^2 = 100\%$

Test for subgroup differences: $Chi^2 = 0.03$, df = 1 (P = 0.87)

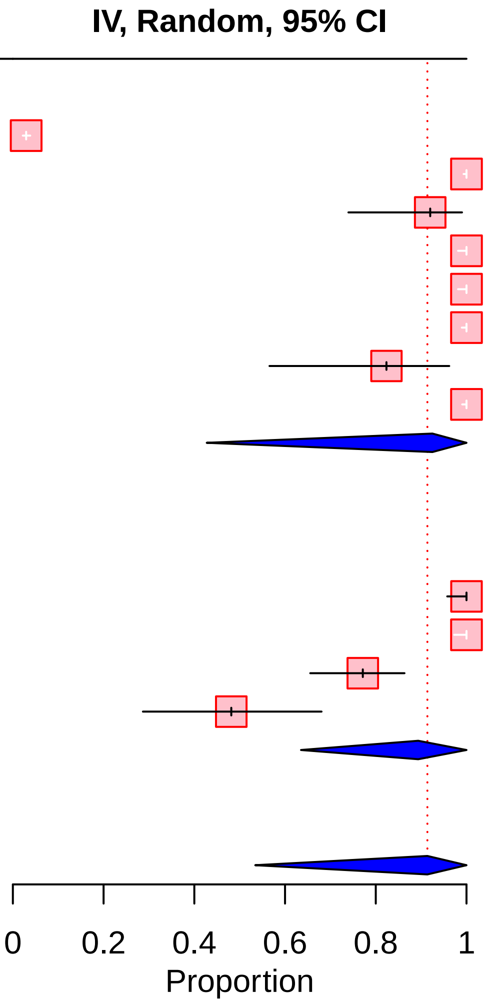

Figure S4: Risk of bias summary of included studies

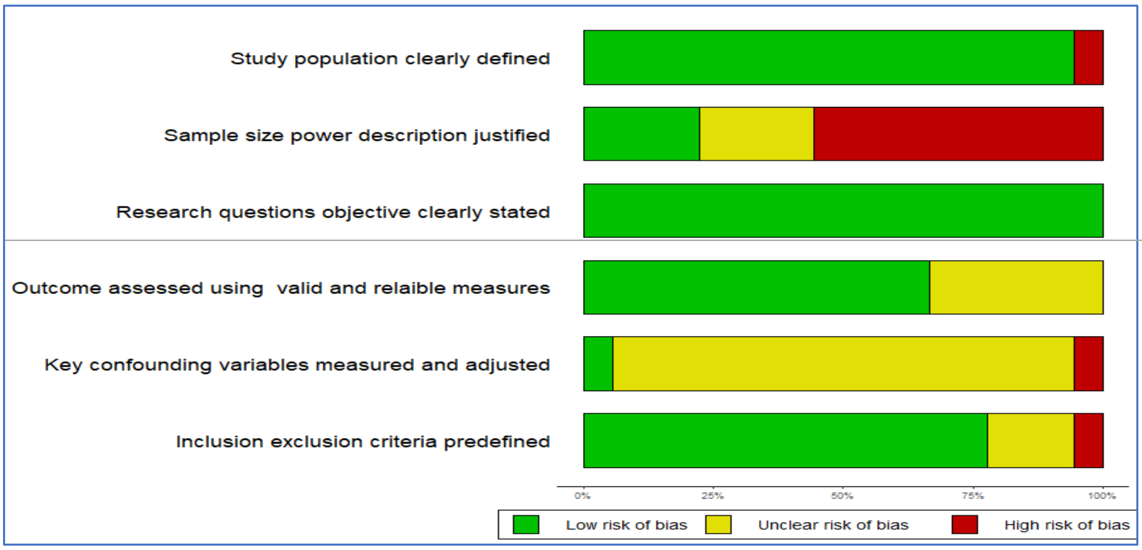

Supplement: Supplementary file 1 [file ijerph-20-05569-s001.zip › ijerph-2291404-supplementary.pdf]
